# Circadian Rhythm in Adipose Tissue: Novel Antioxidant Target for Metabolic and Cardiovascular Diseases

**DOI:** 10.3390/antiox9100968

**Published:** 2020-10-09

**Authors:** Andy W. C. Man, Ning Xia, Huige Li

**Affiliations:** Department of Pharmacology, Johannes Gutenberg University Medical Center, Langenbeckstr, 1, 55131 Mainz, Germany; wingcman@uni-mainz.de (A.W.C.M.); xianing@uni-mainz.de (N.X.)

**Keywords:** adipokines, oxidative stress, sirtuin 1, endothelial nitric oxide synthase, branched-chain amino acids, clock genes

## Abstract

Obesity is a major risk factor for most metabolic and cardiovascular disorders. Adipose tissue is an important endocrine organ that modulates metabolic and cardiovascular health by secreting signaling molecules. Oxidative stress is a common mechanism associated with metabolic and cardiovascular complications including obesity, type 2 diabetes, and hypertension. Oxidative stress can cause adipose tissue dysfunction. Accumulating data from both humans and experimental animal models suggest that adipose tissue function and oxidative stress have an innate connection with the intrinsic biological clock. Circadian clock orchestrates biological processes in adjusting to daily environmental changes according to internal or external cues. Recent studies have identified the genes and molecular pathways exhibiting circadian expression patterns in adipose tissue. Disruption of the circadian rhythmicity has been suggested to augment oxidative stress and aberrate adipose tissue function and metabolism. Therefore, circadian machinery in the adipose tissue may be a novel therapeutic target for the prevention and treatment of metabolic and cardiovascular diseases. In this review, we summarize recent findings on circadian rhythm and oxidative stress in adipose tissue, dissect the key components that play a role in regulating the clock rhythm, oxidative stress and adipose tissue function, and discuss the potential use of antioxidant treatment on metabolic and cardiovascular diseases by targeting the adipose clock.

## 1. Introduction

Obesity is a major public health concern worldwide, and due to its growing frequency, has become a serious financial burden to many countries [1]. Obesity contributes to the pathogenesis of most metabolic and cardiovascular diseases, including diabetes and hypertension [2]. Obesity is characterized by the excess accumulation of fat in adipose tissue, which can result from unhealthy lifestyle such as irregular sleep/wake cycle and high snacking frequency [3]. Adipose tissue is a critical modulator of metabolic and cardiovascular health. There is an increasing consensus that recognize adipose tissue as an important endocrine organ that can alter the metabolism of other distal organs. This is accomplished by the production and secretion of adipocyte-derived cytokines (also known as adipokines). Adipokines have a wide range of signaling and metabolic effects, such as regulating insulin sensitivity, inflammation, and blood pressure [4]. Indeed, adipose function shows strong fluctuation throughout the day. For example, the transportation, storage and metabolism of triglycerides (TGs) or glucose are carried out in the different phases of the day [5]. Adipokine levels show robust oscillations throughout the day [6,7]. These rhythmic oscillations are not mere responses to external stimuli, but are also facilitated by the local clock system that coordinates physiology and external cues. Dysfunction of the adipose tissue could exert deleterious effects on the metabolic and cardiovascular systems.

Oxidative stress is a common pathophysiological event in metabolic and cardiovascular diseases, including obesity, diabetes, and hypertension. Oxidative stress occurs when there is a redox imbalance [8]. Under normal conditions, homeostatic reactive oxygen species (ROS) play important roles as secondary messengers in various intracellular signaling pathways including programmed cell death or necrosis, gene expression regulation, cell signaling cascades activation [9], as well as in both innate and adaptive immune responses [10], and body weight control by modulating satiety and hunger behavior [11]. Oxidative stress can cause adipose tissue dysfunction by stimulating preadipocyte proliferation, adipogenesis and chronic inflammation, which leads to obesity [12]. In obesity, systematic oxidative stress can also be induced by oxidative phosphorylation in mitochondria, superoxide generation from nicotinamide adenine dinucleotide phosphate (NADPH) oxidase (NOX), uncoupled endothelial nitric oxide synthase (eNOS) [13] and reduced antioxidant defense [8,14]. Interestingly, the production of antioxidants and expression of anti-oxidative enzymes have been reported to be regulated by the circadian rhythm [15]. Therefore, oxidative stress seems to have an innate connection with the circadian clock. 

Many organisms have developed an intrinsic mechanism responding to the environmental light-dark cycle. This core timing system ha’s an intimate relationship with many important physiologies and pathways. Circadian (Latin “*circa*” meaning around and “*diem*” meaning a day) clock orchestrates biological processes in adjusting to daily environmental changes. Circadian rhythm generally oscillates in a daily cycle around 24 hours light and dark period in response to abiotic and biotic factors [16]. The circadian clock is composed of molecular clock networks in the core and peripheral tissues. Core circadian clock is located in the hypothalamic suprachiasmatic nuclei (SCN), while peripheral clocks are found in other tissues including the kidney, liver, blood vessels and adipose tissues [17]. The intrinsic circadian clock is autonomous and self-sustaining by the negative feedback loop of molecular clock control. In addition, internal or external factors, which are known as “zeitgeber” (German for time giver), may cue the endogenous circadian cycle and modulate the clock from the molecular to behavioral level [18,19].

Desynchronization of the clock or the misalignment between circadian rhythm and metabolism contributes to the pathogenesis of chronic metabolic and cardiovascular diseases [20]. In experimental models, knockout and mutation in clock genes that lead to the disruption of circadian rhythmicity have revealed the tight link between the circadian clock, adipose tissue function and metabolism [21]. It is estimated that nearly half of all murine genes are expressed in circadian rhythm in at least one tissue of the body [22]. Recently, a circadian transcriptome study in human adipose tissue has shown that 837 transcripts exhibit circadian expression profiles with clear separation of transcripts peaking in the morning (258 probes) and evening (579 probes) [23], suggesting that circadian rhythm in adipose tissue could be a novel therapeutic target for metabolic and cardiovascular diseases.

In this review, we summarize current knowledge on circadian rhythm, adipose tissue function, ROS production and oxidative stress in metabolic and cardiovascular diseases; dissect the key components that play a dual role in regulating the clock rhythm, as well as adipose tissue metabolism, and link adipose circadian rhythms with oxidative stress; and discuss the potential antioxidant treatment of metabolic and cardiovascular diseases by targeting circadian clock in adipose tissue.

## 2. Role of Adipose Tissue in Metabolic and Cardiovascular Health

Adipose tissue has been regarded as a reservoir for energy storage, while currently known as an endocrine organ that regulates energy metabolism and influences systemic metabolic homeostasis through adipokines [21]. In mammals, at least two types of adipose tissue have been described, the white adipose tissue (WAT) and the brown adipose tissue (BAT). These two adipose tissues are originated from different stem cells, and they have distinct localizations, gene expression profiles and functions [24]. In addition, beige adipose tissue is recently described and is located within WAT but shares similar features with BAT [24]. The balance between WAT, BAT, and beige adipocytes is important for maintaining overall energy and lipid metabolism.

### 2.1. White Adipose Tissue

WAT is the most abundant type of adipose tissue and the main energy reservoir in mammals. White adipocytes are characterized by containing a single large unilocular lipid droplet and only a few mitochondria. The primary function of WAT is to synthesize and store excess dietary energy as triacylglycerols (TAGs), and hydrolyze TAG to generate fatty acids during energy deprivation [25]. WAT is also responsible for the secretion of a huge number of hormones and adipokines that regulate energy intake, metabolism and insulin resistance [26,27]. WAT converts excess dietary fats and carbohydrates from the circulation to TAGs by lipogenesis [28]. On the other hand, TAGs are broken down into glycerol and free fatty acids (FFAs) by lipolysis to release and supply the needs of distal organs [28].

### 2.2. Brown Adipose Tissue

Brown adipocytes are characterized by containing multilocular lipid droplets and high mitochondrial density which facilitates thermogenesis [29]. BAT is developmentally different from WAT. BAT originates from early paired box protein 7 (Pax7)^+^ and myogenic factor 5 (Myf5)^+^ embryonic progenitor cells or postnatal myoblasts, while late Pax7 expression is restricted to the skeletal muscle lineage [30,31]. In addition, PR domain containing 16 (Prdm16) is also a known lineage determining factor and transcription cofactor for the development of brown adipocytes [31]. The major activator of BAT is the sympathetic nervous system, signaled by norepinephrine [32]. Upon stimulation, such as cold exposure, BAT is activated to promote non-shivering thermogenesis via uncoupling protein 1 (UCP1), a thermogenic mitochondrial protein that uncouples electron transport from adenosine triphosphate (ATP) production [29]. In addition to its primary role in maintaining body temperature, BAT is also important for energy metabolism, insulin sensitivity, and lipid metabolism due to its high metabolic rate [33,34]. 

### 2.3. Beige Cells and Browning

Recent studies have identified beige adipocytes, which are mitochondria rich and express UCP1, within WAT [35,36]. Beige adipocyte can be considered phenotypically as a fat cell that possesses characteristics between those of the WAT and BAT [37], which indicate that they are energy accumulators that possess thermogenic capacity to dissipate heat through UCP1 [38]. 

The biogenesis of beige adipocytes or the white-to-brown transition of adipocytes is called browning/beiging, which is a temporary adaptive response to external environmental signals. Browning can be stimulated by cold exposure, adrenergic stimulation and treatments with peroxisome proliferation-activated receptor gamma (PPARγ) agonists [39,40], fibroblast growth factor 21 (FGF21) [41], atrial natriuretic peptide (ANP) [42], and bone morphogenetic proteins (BMP) [43]. Browning of adipose tissue has been shown to be beneficial in preventing metabolic and cardiovascular diseases [44]. In addition, cold exposure can stimulate glucose uptake and TG clearance in adipose tissue, which may contribute to the modulation of oxidative stress in adipose tissue [45,46]. Interestingly, FGF21 has recently been shown to have both antihypertrophic and cardioprotective actions [47,48], and has been considered as a promising new therapy target obesity and associated diseases, by activating BAT and by acting on WAT and the liver [49]. Remarkably, FGF21 can regulate circadian behavior and metabolism by acting on the nervous system [50].

### 2.4. Perivascular Adipose Tissue

Apart from the three types of adipose tissue mentioned above, perivascular adipose tissue (PVAT) is also a critical modulator of cardiovascular health [51,52]. PVAT is the ectopic fat depot surrounding most vasculature including large arteries and veins, small and resistance vessels, and skeletal muscle microvessels [52]. This intimate connection between the vascular system and PVAT highlights the importance of PVAT. PVAT controls vascular tone on vessels through releasing various PVAT-derived relaxing factors (PVRFs) [53] and PVAT-derived contracting factors (PVCFs) [54,55]. Moreover, these PVAT-derived factors closely and directly affect vascular homeostatic processes, including vascular inflammation and oxidative stress, vascular tone, smooth muscle proliferation and migration and vascular remodeling [51,56,57].

To date, PVAT has been reported to contain both WAT and BAT, while the white-to-brown ratio is different across the vascular bed. For example, PVATs surrounding larger blood vessels are more BAT-like, whereas those surrounding smaller blood vessels are WAT-like [56,58,59,60]. It is suggested that the adipocytes in PVAT may share a common smooth muscle protein 22 alpha (SM22α^+^) precursors with vascular smooth muscle cells (VSMCs) [61]. During obesity and aging, the gradual shift into WAT-like characteristics of PVAT is associated with the alteration of secretome profile, which may lead to vascular dysfunction, arterial remodeling and increase in blood pressure [62,63]. Apart from cold exposure, exercise training has been shown to induce a BAT-like shift and thermogenic response, which is associated with enhanced eNOS expression and reduced oxidative stress in PVAT [64].

### 2.5. Adipokines

Adipose tissue produces and secretes a variety of bioactive molecules, adipokines, which signals to nearby or remote target organs. Adipose tissue accumulation and altered adipokine profile are linked to chronic inflammation and metabolic and cardiovascular disorders [65]. The primary adipokines that play a role in inflammation, metabolic and cardiovascular diseases include adiponectin, leptin, resistin, visfatin, tumor necrosis factor alpha (TNF-α), interleukin-6(IL-6), plasminogen activator inhibitor-1 (PAI-1), and vascular endothelial growth factor (VEGF) [66,67]. In general, adipokine levels are positively correlated with fat mass, and the functions of adipokines are also related to their site of production. For example, BAT-derived IL-6, an adipokine that are upregulated by proinflammatory stimulation, has been shown to mediate glucose metabolism and energy balance [34]. PVAT-derived adiponectin is an important contributor to vascular function, partly by enhancing eNOS phosphorylation in the endothelium [68]. Notably, most hormones, including adipokines, display daily fluctuation of secretion during the day-night cycle. In addition to the altered adipokine profile, the disruption of this rhythmic secretion of adipokines is also an aggravating factor for the development of metabolic and cardiovascular diseases [69].

## 3. Circadian Rhythm in Adipose Tissue

The intrinsic biological clock of around 24-h cycle that orchestrates the body physiology is controlled by a multi-oscillatory network composed by circadian clocks in most cells and tissues, including the adipose tissue [70]. Clinical studies have reported the circadian pattern and diurnal variation of physiological and pathological cardiovascular events such as heart rate, blood pressure and endothelial function [71,72,73,74,75].

The central circadian is located in the SCN at the anterior hypothalamus, while peripheral clocks are found in the liver, intestine, heart, blood vessel wall and adipose tissue [17,76,77]. In the SCN, photic information transmitted from the retina synchronizes neurons to coordinate circadian outputs [78]. The central clock regulates peripheral clock directly by neuronal or hormonal signaling or indirectly by driving appetite, blood pressure, and body temperature, in order to coordinate circadian gene expression [79,80]. 

The local circadian clocks present in adipocytes regulate many essential adipose tissue processes including lipolysis, adipogenesis, inflammation, browning, thermogenesis, as well as the expression and secretion of adipokines [81]. Therefore, circadian disruption may alter the physiology of adipose tissue and thereby affect whole body energy homeostasis. The rhythmic expression of circadian genes in different white adipose depots in rodents [5,82,83] and humans [84,85,86] have been revealed by both in vitro and in vivo studies. In murine adipose tissue, approximately 10% of genes are expressed in circadian rhythm, which underlines the circadian control over the adipose tissue function [83].

### 3.1. The Molecular Clock Mechanism in Adipose Tissue

At the molecular level, circadian clock in the adipose tissue is based on interlocked transcription–translation feedback loops of clock genes and proteins [87]. These circadian proteins include important transcription factors brain and muscle aryl hydrocarbon receptor nuclear translocator-like protein 1 (BMAL1 a.k.a. ARNTL) and circadian locomotor output cycles kaput (CLOCK), and transcriptional modulator families of Period 1/2/3 (PER1/2/3) and Cryptochrome ½ (CRY1/2) [87,88]. The molecular clockwork in adipose tissue relies on the dimerization of BMAL1 and CLOCK proteins, whereas the heterodimer of CLOCK:BMAL1 binds to E-box sequence (5′-CACGTG-3′) in the promoters and activates the transcription of other circadian genes including *Per1–3* and *Cry1/2* [17]. When PERs and CRYs are expressed in certain amount in the cytoplasm, these proteins dimerize (PERs:CRYs) and translocate into the nucleus, which leads to the inhibition of CLOCK:BMAL1-mediated transcription [17,89].

There are also reinforcing loops that are composed by the circadian nuclear receptors, reverse ERB (REV-ERB α/β) and retinoic acid receptor-related orphan receptors (RORα/β/γ) that control the rhythmic transcription of *Bmal1* and *Clock*. REV-ERBα negatively regulates *Bmal1* and *Clock* expression [90], whereas RORα and RORγ positively regulate *Bmal1* and *Clock* expression via ROR response elements at the promotor regions [91,92]. All the expression patterns of these clock genes exhibit a 24-hour oscillation in adipose tissue [93]. In human adipose tissue, the expression of BMAL1 and CLOCK peaks in the late evening, while the expression of PERs, CRY2, and REV-ERBα peak around late morning [85]. In addition, post-translational modifications also contribute to the regulation of the circadian clock [70,94].

**Figure 1:**
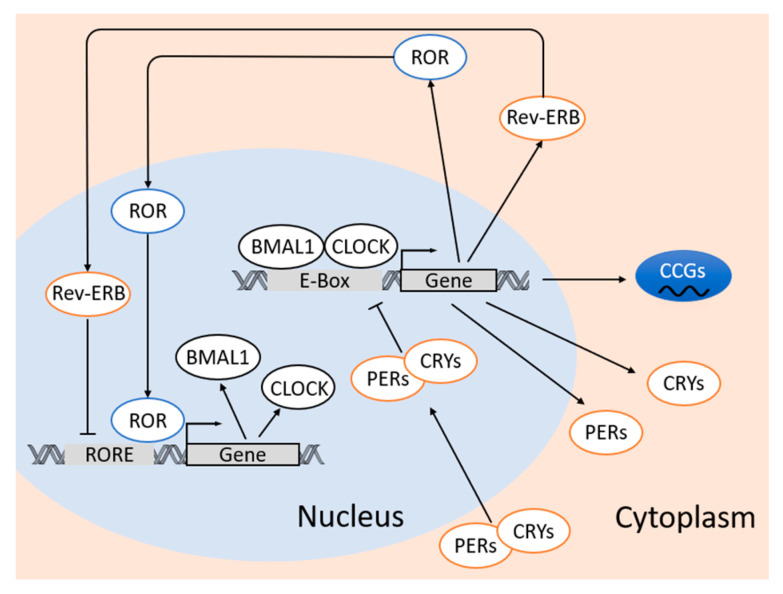
Molecular clock mechanism in adipose tissue. The molecular clockwork in adipose tissue relies on the dimerization of BMAL1 and CLOCK proteins, whereas the heterodimer of CLOCK:BMAL1 binds to E-box sequence (5′-CACGTG-3′) in the promoters and activates the transcription of *Per1–3, Cry1/2, ROR* and *Rev-ERB*. When the cytoplasmic levels of PERs and CRYs reach a certain threshold, PERs and CRYs dimerize and translocate into the nucleus and inhibit the CLOCK:BMAL1-mediated transcription. REV-ERB negatively regulates *Bmal1* and *Clock* expression, whereas RORs positively regulate *Bmal1* and *Clock* expression via ROR response elements (RORE) at the promotor regions. The molecular circadian clock also leads to rhythmic expression of clock-controlled genes (CCGs) through CLOCK:BMAL1-mediated activation of circadian promoter elements including E-boxes, D-boxes, and ROR response elements. BMAL1, brain and muscle Aryl hydrocarbon receptor nuclear translocator--like protein 1; CLOCK, circadian locomotor output cycles kaput; CRY, Cryptochrome; PER, Period; REV-ERB, reverse ERB; ROR, retinoic acid receptor-related orphan receptors.

Apart from the interlocked transcription–translation feedback loop of the clock genes, the oscillation of the circadian clock also leads to rhythmic expression of clock-controlled genes (CCGs) through activation of circadian promoter elements including E-boxes, D-boxes, and ROR response elements [85]. While the same clock machineries are found in most cells, the rhythmic expression of these CCGs are tissue-specific or even cell-type-specific [70] (Figure 1). 

### 3.2. Clock Genes in Adipose Function

Apart from controlling the circadian machinery, circadian clock genes also have important functions in the metabolic and cardiovascular system. In different animal models, circadian clock disruptions generally lead to reduced lifespan, accelerated aging and dramatic effects on body weight gain, adiposity and TG metabolism [95,96,97].

Clock^Δ19^ and Bmal1^−/−^ circadian mutant mice display low and nonrhythmic blood FFA and glycerol levels together with decreased lipolysis rates and increased sensitivity to fasting [5]. Circadian disruption promotes the accumulation of TGs in WAT which leads to increased adiposity and adipocyte hypertrophy [5]. CLOCK and BMAL are also critical in maintaining glucose homeostasis while Clock-mutant mice display phenotype of cardiovascular and metabolic diseases such as obesity and hypertension [7,98]. Deficiency of *Bmal1* leads to impaired adaptive vascular remodeling and increased collagen deposition in the medial layer of blood vessels in mice [99]. Moreover, transcriptome analysis of tissue-specific Clock^Δ19^ mutant mice shows altered regulation of at least 660 genes due to impaired WAT clocks, of which 26% are repressed, while the remaining 74% are induced [95]. A part of these differentially expressed genes is involved in lipogenesis, including those encodes for fatty acid transporters, fatty acid binding protein 9 (FABP9) and glycerol kinase 2 (GK2) [95]. Overexpression of BMAL1 in adipocytes has been shown to increase lipid synthesis activity and promote several factors associated with lipogenesis in vitro [100]. BMAL1 modulates adipogenesis via wingless-type MMTV integration site family (Wnt) signaling in WAT [101], while it exerts transcriptional control on the transforming growth factor-*β* (TGF-β) pathway in BAT. Either global ablation or adipocyte-selective inactivation of BMAL1 increases the mass of BAT and cold tolerance [102]. BMAL1 inhibits brown fat formation through direct transcriptional control of TGF-*β* and bone morphogenetic protein (BMP), which are known to suppress [103] and drive [104] brown adipocyte differentiation, respectively. Activation of TGF-*β*/Smad3 or inhibition of BMP pathways normalizes the enhanced differentiation in *Bmal1*-deficient brown adipocytes [102]. Interesting, antioxidant N-acetyl-L-cysteine can ameliorate symptoms of premature aging in *Bmal1*-deficient mice [105].

REV-ERB and ROR families are important component that link the core clock mechanism with lipid metabolism. REV-ERBs and RORs have been shown to be responsible for adipocyte differentiation [106], lipogenesis, and lipid storage [107,108]. *Rev-erbα*-deficient mice show increased adiposity on both normal chow and high-fat diet (HFD) as a result of increased fat uptake by adipose tissue [109]. REV-ERB agonist treatment has been shown to reduce fat mass and alleviate dyslipidemia and hyperglycemia in obese mice [110]. REV-ERB agonist can suppress orexinergic gene expression, while *Rev-erbβ*-deficient mice have increased orexinergic transcripts, suggesting the involvement of REV-ERB in regulating appetite [111]. On the other hand, REV-ERB*α* appears to have an opposite role on adiposity in BAT. Deletion of *Rev-erbα* results in an improved thermogenic response to cold challenge, while downregulation of *Rev-erbα* leads to the induction of UCP1 expression [112]. As opposed to BMAL1, REV-ERB*α* inhibits key components of the TGF-*β* signaling pathway [113], thereby promotes BAT development and adipogenesis.

*Per2*^−/−^ mice displays altered lipid metabolism, with drastic reduction of total TAGs and non-esterified fatty acids [114]. PER2 represses PPARγ-mediated transcriptions, thereby altering the expression of genes involved in adipogenesis, insulin sensitivity and inflammatory response [114]. Moreover, lack of PER2 promotes adipocyte differentiation in cultured fibroblasts [114]. PER3 has been reported to modulate adipogenesis by regulating Krüppel-like factor 15 (KLF15) expression, while deletion of *Per3* promotes adipogenesis in vivo [115]. *Cry1^−/−^Cry2^−/−^* mice have reduced body weight and WAT due to higher energy expenditure and heat production compared with wild-type controls under normal chow [116]. *Cry1^−/−^Cry2^−/−^* mice also have an increased sensitivity to HFD-induced obesity, which is associated with increased insulin secretion, elevated lipogenesis and TG accumulation in WAT [117] and augmented proinflammatory cytokine levels [118]. In addition, meta-analysis indicates that genetic variants at *Cry1* are associated with insulin resistance and diabetes risk in human [119].

To date, only a few studies have reported the phenotypes and effects on adipose tissue function and oxidative stress in adipose tissue-specific clock gene knockout mice (Table 1). Adipocyte-specific deletion of *Bmal1* results in obesity in mice with a shift in diurnal rhythm of food intake and loss of circadian variation in plasma TG and glucose levels [120]. Deletion of the adipocyte *Bmal1* is associated with a reduced number of polyunsaturated fatty acids in adipocyte triglycerides, and loss of rhythmic expression of clock and clock-output genes in both BAT and WAT [120]. In addition, adipocyte-specific *Clock*^Δ19^ mutation increases body weight and fat mass in mice [121]. The young mortality rates of these mice are increased and the rates of glucose tolerance are reduced compared to wild type mice [121]. Moreover, brown adipocyte-selective *Bmal1*-deficient mice have reduced blood pressure during resting phase, associated with reduced PVAT-induced vasoconstriction [122]. Indeed, angiotensinogen mRNA and angiotensin II levels in PVAT of brown adipocyte-selective *Bmal1*-deficient mice are significantly reduced [122]. These studies suggest that clock aberration specifically in adipose tissue can result in significant alterations of the cardiovascular and metabolic health. Circadian clock genes in adipose tissues are important in maintaining the robust relationship between circadian rhythm and metabolic and cardiovascular health. However, most of the observations currently made are based on animal models with global ablation or mutants of circadian clock genes, the contribution of these circadian clock genes in adipose tissue function warrants further studies by using adipose tissue-specific knockout/mutant mice. 

## 4. Oxidative Stress and Circadian Rhythm

Generation of ROS is a normal physiological process in all aerobic organisms. Under normal conditions, deleterious ROS are mostly removed by the cellular antioxidant systems. Oxidative stress occurs when the production of oxidant molecules, such as superoxide (^•^O^−^_2_) and hydrogen peroxide (H_2_O_2_), exceeds the capacity of antioxidants, including catalase, glutathione peroxidase (GPx), superoxide dismutase (SOD), NAPDH and peroxisomes to defend against these insults. Excessive ROS are known to cause lipid peroxidation and oxidative modifications of proteins and nucleic acids that cause metabolic and cardiovascular complications [123,124,125,126]. 

In obese animals and patients, adipocytes become hypertrophic leading to hypoxia [52]. The expression of the key mediator of hypoxia, hypoxia-inducible factor alpha (HIF-1α) is augmented in the adipose tissue of obese subjects [127]. HIF-1α stimulates the production of inflammatory mediator, such as TNF-α and IL-6, and suppresses the expression of adiponectin [128]. HIF-1α is interconnected to the circadian clock and has been shown to regulate clock gene expression by direct promotor binding, while CRY1/2 stabilize HIF-1α in response to hypoxia [129,130]. This suggests a bidirectional interaction between hypoxia signaling pathway and the circadian clock. Obesity-induced adipocyte hypertrophy is associated with reduced insulin sensitivity, increased oxidative stress, and abnormal adipokine secretory profile, including an elevated array of other pro-inflammatory factors [IL-1β, IL-8, resistin and monocyte chemoattractant protein 1 (MCP1)] and a reduced array of anti-inflammatory factors (IL-10, adiponectin and FGF21) [131,132]. These inflamed WATs become severely dysfunctional and fail to store surplus energy, which lead to ectopic fat deposition in other metabolic tissues such as liver and skeletal muscle [133]. Therefore, obesity engenders nutrient stress that alters mitochondrial function, while the elevated mitochondrial substrate load augments electron transport chain activity and ROS production [134]. The increased oxidative stress in adipose tissue alters the secretion of adipokines and leads to metabolic and cardiovascular complications [51,134]. As mentioned, secretion of adipokines is in circadian rhythm [135]. Increased secretion of IL-6 is associated with excessive sleeping at day cycle and fatigue, while a reduction of IL-6 secretion is associated with sufficient sleeping at night cycle, whereas both TNF-α and IL-6 secretions have been reported to increase in sleep apnea independently of obesity [136]. Indeed, the susceptibility of wild-type mice to HFD-induced obesity depends on the time of feeding [137]. Mice fed with HFD late in their activity period are more susceptible to induce obesity while mice fed with HFD early in the activity period are resistant to obesity [137]. This suggests that the pathogenesis of metabolic and cardiovascular diseases may not solely be due to excessive nutrient-induced obesity, while circadian dysregulation of metabolism and oxidative stress may be the key components that drives obesity.

The cellular redox state in plants and animals has been known to oscillate over circadian time [138]. The expression of major antioxidant enzymes is rhythmic, but there are high divergency of amplitude, phasing, and presumable protective value among different tissues [15]. *Bmal1*^−/−^ mice are reported to have increased ROS levels in different tissues and show symptoms of premature aging [97]. Apart from obesity, people with sleeping disorder or night shift workers also show augmented levels of oxidative stress damages due to a reduced antioxidant defenses [139,140], which may contribute to the increased risk of metabolic and cardiovascular diseases. These results suggest an intrinsic relationship between oxidative stress, circadian rhythm, and adipose tissue function.

**Figure 2:**
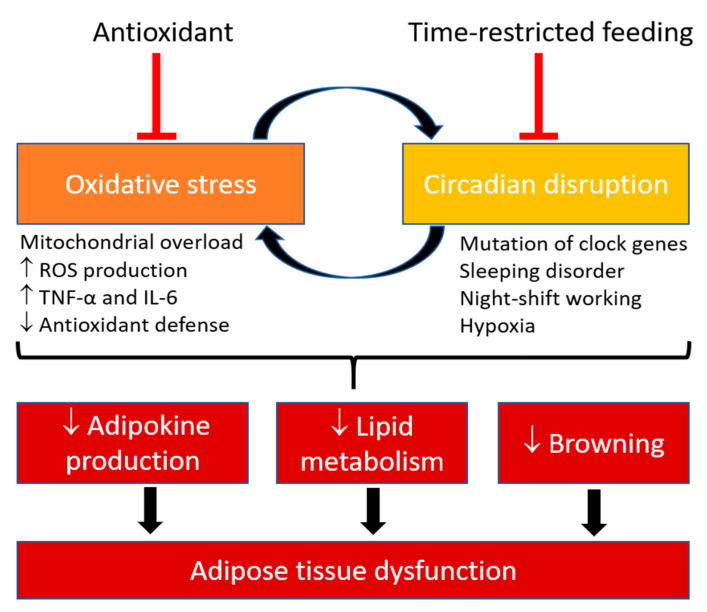
Oxidative stress and circadian rhythm are interrelated in modulating adipose tissue function. Circadian clock regulates the rhythmic oscillation of cellular redox function, while changes in redox state in the cell can influence the clock machinery. Oxidative stress occurs when the production of oxidant molecules exceeds the capacity of antioxidants to defend against these insults. The cellular redox state has been known to oscillate over circadian. Animal models of circadian disruption by hypoxia, clock genes knockout or mutant, night-shift workers and patients with sleeping disorder are reported to have increased oxidative stress and reduced antioxidant defence. Disruption of circadian and increased oxidative stress lead to reduction of adipokine productions, lipid metabolism and browning process in adipose tissue. Metabolic and cardiovascular diseases that are caused by disruption of circadian rhythm and increased oxidative stress can be prevented by antioxidants treatment and time-restricted feeding. ROS, reactive oxygen species; TNF-α, Tumor necrosis factor alpha; IL-6, interleukin 6.

Recently, considerable evidence has been shown to support the hypothesis that oxidative stress and circadian rhythm are interrelated, as the circadian clock regulates the rhythmic oscillation of cellular redox function, while changes in redox state in the cell can influence the clock machinery [141] (Figure 2). Indeed, oxidative stress can drive the expression of REV-ERBα, which in turn regulates the expression of antioxidant transcription factor forkhead box protein O-1 (FOXO1) as well as stimulates autophagy and mitochondrial biogenesis [142]. Circadian oscillation of H_2_O_2_ level is observed in mammalian cells and mouse liver, and can directly regulate CLOCK circadian function via cysteine oxidation [143]. Moreover, nicotinamide adenine dinucleotide (NAD) has been recently recognized as a “metabolic oscillator” that displays circadian oscillations and is the missing-link between energy metabolism and circadian control [144,145,146]. Inhibition of catalase results in a lengthened period of the *Per1* gene oscillation and hypoxia leads to a higher amplitude oscillation of NAD+/NADH ratio and a highly oxidized cellular environment during the night [147]. The DNA binding activity of CLOCK:BMAL1 heterodimer is dependent on the cellular redox status (NADH/NAD and NADPH/NADP ratios) [148], while NADPH/NADP ratio oscillates in a circadian manner [147]. Pentose phosphate pathway (PPP) can regulate the circadian clock via NADPH metabolism [149]. Inhibition of PPP has been shown to increase oxidative stress, lengthen circadian period, activate nuclear factor erythroid 2-related factor 2 (NRF2) pathway, increase CLOCK:BMAL1 DNA binding and alter CCGs expression in vitro [149]. In addition, NRF2 pathway appears to regulate clock function, as *Nrf*^−/−^ cells have flattened clock gene expression and blunted *Per2* circadian expression [150].

Recently, thermogenic effect of ROS has been reported. Increased adipocyte ROS levels and oxidative stress can promote thermogenesis in adipocyte tissue [151,152]. Indeed, activation of thermogenesis in murine BAT by either cold exposure or β-adrenergic stimulation is associated with an elevated levels of mitochondrial superoxide, H_2_O_2_, and lipid hydroperoxides [152]. The thermogenic action of mitochondrial ROS is mediated through protein cysteine modification [152]. Moreover, the substantial and selective accumulation of succinate, a mitochondrial metabolite, may be a potent source for thermogenic ROS in brown and beige fat [151].

Another evidence that links circadian rhythm and oxidative stress is the experimental results from the use of antioxidants to entrain the clock. Melatonin is a well-known hormone involved in circadian rhythm regulation and contributed to protection against oxidative stress [153]. Indeed, melatonin is a direct free radical scavenger, which has anti-inflammatory and antioxidant effects, due to its electron-rich aromatic indole ring [154,155]. Recently, melatonin treatment has been shown to stimulate thermogenesis of BAT in response to cold exposure via activating citrate synthase and respiratory chain complexes I and IV in diabetic and obese rats [156]. Moreover, melatonin can promote the differentiation of white adipocytes into the beige adipocytes and improve adipose tissue function [157]. These experiments suggest that potential use of antioxidants to entrain the circadian clock.

### 4.1. Effects of Time-Restricted Feeding and Intermittent Fasting on Circadian Rhythm and Metabolism

Feeding and fasting patterns have been shown to drive daily rhythms in the activities of key energy regulators including adenosine 5′-monophosphate–activated protein kinase (AMPK), cAMP-response element binding protein (CREB) and protein kinase B (AKT) [158,159], which suggest a clear connection between circadian rhythm and metabolism. Constant light causes disruptions in the rhythmic oscillation of plasma levels of glucose and TG in mice and eliminations of the circadian expression of genes involved in lipid metabolism in WAT, which can be restored by time-restricted feeding to various degrees [160]. This suggests that food intake is an important zeitgeber, which may be stronger than light-dark cycle, that regulates lipid metabolism in WAT. Feeding and restrictive meal timing have been shown to modulate circadian rhythm and generate pronounced phase shifts in circadian gene expression in different peripheral tissues [161,162]. Temporally restricted feeding in mice alters the circadian expression profile in BAT, inguinal WAT and epididymal WAT [83]. In rat, either wrong time restricted feeding or high-fat-high-sugar diet disturbs the peripheral clock rhythmicity, and alters *Ucp1* and *PPARγ coactivator 1 alpha* (*Pgc-1α)* expression in BAT [163]. In mice, time restricted feeding attenuates obesity, hyperinsulinemia and inflammation, associated with improvements in circadian clock oscillation, and mTOR and AMPK pathway function [158]. In a recent study, time restricted feeding has been shown to prevent obesity and improve metabolic function in whole-body *Cry1;Cry2* knockout and in liver-specific *Bmal1* and *Rev-erba/b* knockout mice [164]. 

Intermittent fasting is an dietary pattern in which minimal calories are consumed 1 to 4 days per week, followed by *ad libitum* diet on the remaining days [165], and has been shown to reduce the risk for metabolic diseases in human [166,167,168]. Intermittent fasting has been shown to decrease energy intake, body weight, adiposity, and improve glucose tolerance in HFD-fed mice [169]. HFD-induced adipose tissue inflammation and fibrosis can also be ameliorated by intermittent fasting [169]. Diurnal intermittent fasting during Ramadan can reduce inflammation and oxidative stress [170], and affect circadian rhythms of energy expenditure and body temperature in healthy people [171]. In addition, Ramadan intermittent fasting ameliorates the expression of antioxidants (*Sod2, and Nrf2*), and metabolic regulatory genes (*Sirt1* and *Sirt3*) in obese and overweight subjects [172]. A recent randomized clinical study has suggested the intermittent fasting-medicated alteration of peripheral clock genes expression in subcutaneous adipose tissue in obese women, despite intermittent fasting does not uniformly impact the expression of clock genes [173]. Nevertheless, studies on time-restricted feeding and intermittent fasting have provided strong evidence that connects circadian clock and energy metabolism.

### 4.2. Clock-Controlled Pathways in Adipose Tissue

As mentioned, the molecular clock machinery drives the circadian transcription of CCGs in peripheral tissues. These CCGs encode for many important regulatory proteins and enzymes that involve in different vital cellular processes. The circadian regulation of CCGs, however, is highly different among tissues, which complicates the research field of circadian rhythm. To date, there are only few studies focused on the circadian regulation in adipose tissue. Identification of important CGGs and pathways involved in adipose tissue function is important to improve the understanding of clock involvement in the pathophysiology of metabolic diseases. Here, we propose some important targets and summarize important findings that link circadian rhythm and oxidative stress in adipose tissue.

#### 4.2.1. Peroxisome Proliferator–Activated Receptors

PPARs are ligand-activated transcription factors of nuclear receptor superfamily consisting of three subtypes (PPAR*α*, PPAR*β*/*δ*, and PPAR*γ*), which are differentially expressed among tissues in mammals [174]. PPAR family plays a major role in regulating energy homeostasis and metabolic function, via controlling the transcription of genes involved in lipid and glucose metabolism [175,176]. PPAR*α* is abundant in BAT, whereas PPAR*γ* is expressed in both BAT and WAT [177]. *Pparα* and *Pparγ* mRNA expression are maximal in WAT at the end of the inactive phase, which prepare the metabolic machinery to metabolize fat [178]. PPAR*β*/*δ* promotes lipolysis by activating genes involved in fatty acid oxidation in adipocytes [179]. Expression of PPAR*β*/*δ* has been shown to oscillate in-phase with that of UCP1 [178], suggesting that PPAR*β*/*δ* could regulate the circadian control of energy dissipation in BAT. PPARγ and PPARα synergize to induce browning of WAT in vivo, via PPARγ activation and PPARα-mediated FGF21 expression [180].

In clinical practice, PPAR*α* agonists are proven to reduce low density lipoproteins (LDLs) and TGs, whereas PPAR*γ* ligands are used to improve glycemic control via insulin sensitization in patients with type 2 diabetes [181]. In obese rats, PPARγ agonists also increase insulin sensitivity, reduce fasting insulin levels and TG concentration, and increase hydrogen sulphide (H_2_S) production in PVAT, which improves in the anticontractile effect of PVAT on aortic rings [182]. H_2_S activates PPARγ by sulphhydrating cysteine 139 residue in PPARγ leading to enhancement of glucose uptake and lipid storage [183]. 

The rhythmic expression of PPARs is mediated by the CLOCK:BMAL heterodimer [184]. On the other hand, PPAR*α* activates *Bmal1* expression by binding to the peroxisome-proliferator response element (PPRE) at its promotor region [185,186]. Obesity attenuates expression of BMAL1and REV-ERB [187], which are correlated with increased expression of PPAR*α* [16]. *Pparγ*-deficient mice display dampened behavioral and cellular circadian rhythms [188], while PPAR*α* agonists have been shown to promote locomotor activity and feeding daily rhythms in mice [189]. In addition, PPAR*α* agonist, bezafibrate, has been shown to normalize *Per2*, *Bmal1*, and *Rev-erbα* circadian expression in adipose tissue of mice with circadian disruption [190]. Indeed, many PPARγ target genes, including *Adiponectin* and *Leptin,* show rhythmic expression in WAT [178]. Moreover, PPARγ pathway also regulates important antioxidant enzymes including heme oxygenase-1 (HO-1) and NRF2 [191], suggesting the possible link between circadian rhythm and oxidative stress.

#### 4.2.2. PPARγ Coactivator 1

The PGC1 is a family of transcriptional coactivators that regulates mitochondrial biogenesis. PPARγ and PGC-1α are important molecules that regulate mitochondrial and adipose function by modulating the white-to-brown differentiation of adipocytes [192]. PGC-1α expression can be induced in BAT during cold exposure [193]. 

All family members of PGC1 exhibit circadian expression, while PGC1*α* can in turn stimulate the expression of *Bmal1*, *Clock*, *Per2*, and *Rev-erbα* through coactivation of the ROR family [194,195]. *Pgc-1α* null mice have been shown to have disrupted clock and metabolic genes expression, which leads to abnormal diurnal rhythms of body temperature, activity, and metabolism [194]. On the other hand, *Pgc-1β* null mice display altered expression of various nuclear-encoded genes that are responsible for mitochondrial and metabolic functions in BAT, while HFD-feeding induces hepatic steatosis and increases serum TG and cholesterol levels in these mice [196], suggesting that PGC-1β plays an important role in controlling mitochondrial oxidative energy metabolism.

#### 4.2.3. SIRT1

Sirtuin 1 (SIRT1) belongs to the family of nicotinamide adenine dinucleotide (NAD)-dependent protein deacetylase [197]. SIRT1 is involved in many biological processes, including cell proliferation and survival, DNA damage repairment and energy metabolism [198,199], which is an important therapeutic target for metabolic and cardiovascular diseases [200]. 

SIRT1 plays a pivotal role in reducing local superoxide production, modulating browning process, and promoting adipokines production in adipose tissue [201]. In adipose tissue-specific *Sirt1*-deficienct mice, the obesity-induced brown-to-white transition of PVAT is exaggerated in vivo, which augments endothelial dysfunction [201]. In obese mice, treatment with SRT1720 (a SIRT1 specific activator) prolongs the lifespan and reverses obesity-induced organ damages via normalizing the acetylation of PGC-1α to promote mitochondrial biogenesis in adipose tissue [202]. Activation of SIRT1 can normalize inflammatory insult-induced [203] or oxidative stress-induced adipokines dysregulation from adipose tissue and prevent arterial remodeling [204,205]. Moreover, resveratrol, a polyphenol that can activate SIRT1, can ameliorate adipokine release from dysregulated PVAT via the SIRT1/AMPK pathway [206]. In adipocytes, SIRT1 regulates the secretion of adiponectin through the interaction with FOXO1 [206].

In addition, SIRT1 is involved in the crosstalk between the circadian clock and energy metabolism [207]. SIRT1 is required for high-magnitude circadian transcription of several core clock genes [208], and regulates the transcription of other circadian-related genes by deacetylation of histone H3 lysine 9 (H3K9) on their promoters [209]. SIRT1 deacetylates BMAL1 and PER2 to affect their activities [210,211]. In addition, a negative reciprocal relationship is reported between SIRT1 and PER2, where PER2 negatively regulates the transcription of *Sirt1* through binding to the CLOCK:BMAL1 binding site at *Sirt1* promoter [211]. In *Sirt1*-deficienct mice, disrupted circadian rhythms and altered amplitude of *Per1*, *Per2*, *Cry1* and *Cry2* expression are observed [211].

One of the targets of CLOCK:BMAL1 heterodimer is nicotinamide phosphoribosyltransferase (NAMPT), an rate-limiting enzyme in the biosynthesis of NAD which can regulate SIRT1 activity in circadian rhythm [212,213]. As aforementioned, NAD also plays a pivotal role in circadian epigenomic regulation and shows robust diurnal rhythms [209]. High NAD level inhibits the DNA binding activity of CLOCK:BMAL1 [148]. While NAD level affects the activity of SIRT1, SIRT1-mediated deacetylation of acetyl-CoA synthetase 1 (AceCS1) affects the circadian levels of metabolites including NAD and acetyl-CoA [214]. Indeed, the circadian level of acetyl-CoA may also lead to the circadian acetylation of histones and other non-histone proteins [215,216]. These suggest that SIRT1 is a potent regulator that links circadian, oxidative stress and cellular energy metabolism.

#### 4.2.4. BCAA Metabolism and mTOR Signaling

Recent transcriptome studies in human adipose tissue reveal the circadian regulations of lipid and nucleic acid metabolism, as well as key metabolic pathways such as the citric acid cycle and branched chain amino acid (BCAA) degradation [23]. High levels of BCAAs have been recently identified as contributors of chronic inflammation and lead to the development of insulin resistance and diabetes [217]. BCAAs may also trigger the production of ROS through the activation of NOX1 and NOX2 [218].

Mammalian target of rapamycin (mTOR) is an important regulator of eukaryotic growth and metabolism, sensor of cellular nutrient and energy levels, and is involved in protein synthesis [219,220]. BCAA can activate mTOR, while mTOR is also particularly sensitive to BCAAs. BCAAs are required for cell proliferation in a mTOR complex 1 (mTORC1)-dependent pathway [221]. mTOR signaling links the circadian clock and cellular metabolism. In the SCN, mTOR pathway can be modulated by light-dark cycle [222]. Moreover, protein 70 S6 kinase 1 (S6K1), a key factor in the mTOR pathway, is able to phosphorylate BMAL1 in circadian rhythm, thus affects the CLOCK:BMAL1-mediated circadian translational machinery [223]. In mouse adipose tissue, mRNA expression of Rictor and mTOR peaks during the day and troughs at night, while Rictor-adipose-tissue-specific KO mice have altered circadian genes expression in adipose tissue and non-dipping hypertension development [224,225]. 

In addition, BCAA is a negative regulator of KLF15 expression at transcriptional level via phosphoinositide 3-kinases (P13K)/AKT signaling [226]. Adipose KLF15 has been recently identified as an essential regulator of adipocyte lipid metabolism and systemic energy balance. Mice lacking KLF15 in adipose tissue have decreased adiposity and are protected from HFD-induced obesity [227]. KLF15 regulates key genes responsible for TG synthesis and inhibits lipolytic action [227]. This suggests that BCAA metabolism and mTOR signaling in adipose tissue are critical targets in modulating circadian rhythm and cardiovascular function.

#### 4.2.5. GLP-1 and DPP-IV

The enteroendocrine incretin hormone glucagon-like petide-1 (GLP-1) has been reported to have protective effects on the cardiovascular system [228] and to improve endothelial function in obesity [229]. It is previously reported that the secretion of GLP-1 is in a circadian-manner [230,231]. GLP-1 has been shown to activate genes related to fatty acid oxidation and insulin signaling pathways, thus enhancing antioxidant capacity through its interaction with the G protein-coupled receptor GLP-1R in adipose tissue [232]. GLP-1 receptor agonist, liraglutide, has been shown to alleviate vascular dysfunction by modulating the protein kinase A (PKA)-AMPK-PGC-1α pathway and enhancing antioxidant enzymatic system Nrf2/HO-1 in PVAT of HFD-induced obese mice [233].

Dipeptidyl peptidase IV (DPP-IV) is a serine protease responsible for cleaving and inactivating of incretin peptides and GLP-1, which is correlated to the progression of metabolic and cardiovascular disease [234]. Various DPP-IV inhibitors have been shown to exert direct antioxidant effects, increase insulin secretion and improve glycemic control in diabetic animals and humans [235,236,237]. DPP-IV inhibitor, teneligliptin, reduces atherosclerosis progression in *apolipoprotein E* (*ApoE*) knockout mice by alleviating inflammation and oxidative stress in both the vasculature and PVAT [238]. Indeed, DPP-IV inhibition seems to result in an overall increase in GLP-1 levels with preserved circadian rhythm throughout the day [239]. Recently, treatment with vildagliptin, another DPP-IV inhibitor, has been shown to restore the normal dipping pattern of blood pressure in rat model of salt-dependent hypertension [240]. These results indicate DPP-IV as a potential treatment target for circadian disruption and adipose dysfunction.

#### 4.2.6. AMPK

AMPK is a cellular energy sensor whose phosphorylation activates cellular catabolism [241]. AMPK consists of a catalytic (α) subunit and two regulatory (β, γ) subunits. The AMPKα1 isoform is expressed in adipose tissue [242]. Adipose tissue-specific AMPKα1/α2 KO mice display impaired mitochondrial integrity and biogenesis and reduced thermogenic gene expression in adipose tissue upon cold exposure [243], suggesting a role of AMPK in regulating the browning process of WAT. On the other hand, obesity-linked PVAT dysfunction is associated with AMPK phosphorylation [244].

AMPK is an important energy sensor that connects nutrient signal to circadian clocks. Circadian oscillations in the phosphorylation of the AMPK substrates acetyl-coA carboxylase (ACC) and Raptor have been observed [245,246], suggesting the circadian activity of AMPK. AMPK also phosphorylates CRY1 at Ser 71 residue, which stimulates the direct binding of the F-box and leucine-rich repeat protein 3 (FBXL3) to CRY1 for ubiquitin-mediated degradation [247]. Moreover, AMPK phosphorylates and activates casein kinase Iε, which leads to degradation of PERs [248]. Indeed, pharmacological activation of AMPK has been shown to induce a phase shift in circadian rhythm in a tissue-specific manner [249,250,251]. *Ampk1*-deficient mice have attenuated diurnal alteration of core body temperature, altered circadian gene expression in adipose tissue, and blunted NAMPT rhythmicity [242]. These results suggest that AMPK is an important metabolic cue to entrain peripheral clocks. However, further studies are warranted to dissect the precise role of AMPK in the entrainment of adipose tissue clocks.

#### 4.2.7. eNOS

Endogenous NO generated from eNOS is critical in modulating endothelial function and homeostasis. eNOS is also involved in other vascular processes including leukocyte adhesion, smooth muscle cell proliferation and migration, and platelet aggregation [252]. In addition, eNOS defends against oxidative stress by producing NO which can enhance superoxide dismutation by stimulating SOD expression [253,254]. The beneficial effects of important crosstalk between SIRT1 and eNOS in the endothelial function and oxidative stress defense has been reported extensively [200,255,256].

There is evidence showing that the activities of eNOS are regulated by the peripheral circadian clock in different tissues including brain, aorta and lung [257,258,259]. Impaired eNOS signaling has been reported in mice with circadian disruption [99,260,261], although eNOS expression is not altered in these mice. It has been shown that post-translational mechanisms, especially phosphorylation of eNOS activity, are compromised, consistent with observations demonstrating that eNOS activity exhibits a circadian variation [99,260,262]. eNOS uncoupling and biopterin imbalance have been reported in *Bmal1*-KO mice [263]. Interestingly, leptin has been shown to control the circadian release of NO via TNF-α signaling [264]. This suggests that the circadian activity of adipose eNOS may be regulated via post-translational modification but not by transcriptional control.

The involvement of eNOS in circadian regulation is controversial. Indeed, eNOS may not involves in the photic entrainment in mice, evidenced by no significant changes in phase-shift locomotor activity [265] and circadian blood pressure [266] in *eNOS*^−/−^ mice compared to wild type mice during light-dark or dark-dark cycle. On the other hand, impaired circadian rhythmicity is related to a decrease of NO production with aging [260]. NO has been shown to upregulate the circadian expression of *Per* genes via the cAMP response element–dependent and the E-box enhancer element–dependent pathways [260]. Yet, NO cannot improve the circadian variation in blood pressure of circadian disrupted mice [260]. These suggest that eNOS may not be an important player in modulation the central circadian clock. Nevertheless, the role of eNOS in adipose clock remains to be elucidated, but likely via NO production.

**Figure 3:**
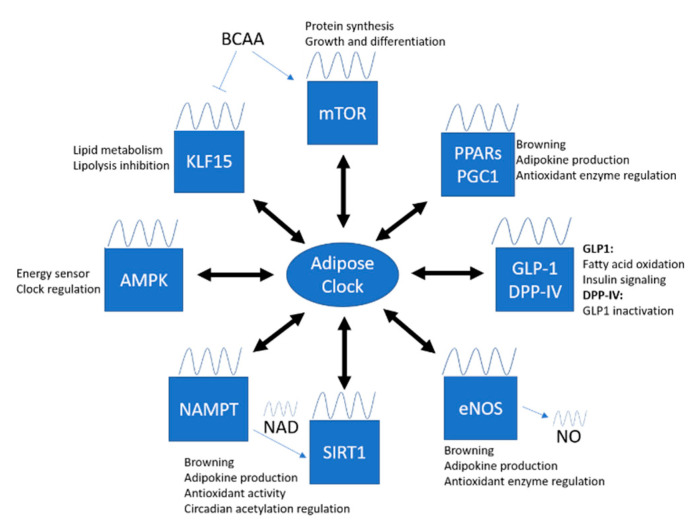
Crosstalk between adipose tissue clock and important signaling targets. Considerable evidence has been shown to support the hypothesis that oxidative stress and circadian rhythm are interrelated. Identification of important CGGs and pathway involved in adipose tissue function is important to improve the understanding of clock involvement in the pathophysiology of metabolic diseases. The intact circadian oscillations of these targets are important in maintaining the balance between oxidative status and energy metabolism in the adipose tissue. mTOR, mammalian target of rapamycin; PPARs, peroxisome proliferator-activated receptors; PGC1, PPARγ coactivator 1; GLP-1, glucagon-like petide-1; DPP-IV, dipeptidyl peptidase-IV; eNOS, endothelial nitric oxide synthase; NO, nitric oxide; SIRT1, sirtuin 1; NAD; nicotinamide adenine dinucleotide; NAMPT; nicotinamide phosphoribosyltransferase; AMPK, 5’ adenosine monophosphate-activated protein kinase; KLF15, Krüppel-like factor 15; BCAA, branched-chain amino acid.

Recently, both gene and protein expression of eNOS have been detected in adipose tissues [267,268], while eNOS in PVAT contributes to vascular NO production [269,270,271]. eNOS is abundantly found in both isolated brown adipocytes and BAT [272], while eNOS-derived NO can promote adiponectin synthesis and mitochondrial biogenesis [273]. On the other hand, HFD-induced PVAT dysfunction is associated with reduction of eNOS expression and NO production [274]. Treatment with an anti-inflammatory and antioxidant drug, methotrexate, has been shown to improve PVAT dysfunction and ameliorate adipokine dysregulation via the activation of the AMPK/eNOS pathway [275]. These suggest the importance of eNOS and NO in modulating adipose tissue function, browning and thermogenesis. Thereby, dissecting the crosstalk between eNOS and the local clock in adipose tissue could be an interesting direction for future studies (Figure 3).

## 5. Conclusions

Obesity is characterized by excess accumulation of fat in adipose tissue, which leads to adipocyte hypertrophy and hypoxia. The effects of excess energy consumption, overloading of mitochondrial activity and generation of oxidative stress in adipose tissue are previously known to associate with obesity. However, results from time-restricted-feeding experiments have shown that circadian rhythm is a critical component that can alter adipose tissue function. Misalignment between circadian rhythm and metabolism contributes to the pathogenesis of chronic metabolic and cardiovascular diseases. Disruption of the circadian rhythmicity is associated with augmented oxidative stress and aberrated adipose tissue function and metabolism.

In experimental models, knockouts or mutations in clock genes that lead to the disruption of circadian rhythmicity have suggested a tight link between the circadian clock, adipose tissue function and metabolism. However, since peripheral clock machinery is highly tissue-specific, there is a need to generate adipose tissue-specific knockout mice to dissect the adipose clock. To date, there are only a few studies that generated adipose tissue-specific clock gene knockout mice and reported the effects on adipose tissue function and oxidative stress (Table 1). Further in vivo studies using these adipose tissue-specific knockout mice are needed to dissect the detailed crosstalk between the clock and oxidative stress in adipose tissue. 

Considerable evidence supports the hypothesis that oxidative stress and circadian rhythm are interrelated, as the circadian clock regulates the rhythmic oscillation of cellular redox function, while changes in redox state in the cell can drive the clock machinery. This gives rise to the use of potential antioxidant treatment to target and entrain the adipose tissue clock. Although the detailed mechanisms and targets of CCGs in adipose tissue are not completely known, we have summarized some potential molecular targets in adipose tissue for antioxidant treatment. The uses of antioxidant drugs, for example, liraglutide (GLP-1 receptor agonist), rosiglitazone (PPARγ agonist), SRT1720 (SIRT1 activator), as well as antioxidant food supplements including polyphenols can also retrain circadian clock and adipose tissue function in human. However, these warrant further systematic in vivo studies to explore the best treatment/prevention strategies.

## Figures and Tables

**Table 1:** Studies using adipocyte-specific clock genes mutant/knockout mice.

Gene Mutation/Knockout	Mice Phenotype	Effect on Circadian Rhythm and Oxidative Stress	Refs
*Bmal1*^−/−^ driven by *aP2* promoter	↑ HFD-induced obesity ↑ HFD-induced adiposity ↑ plasma leptin level ↓ leptin signaling ↓ energy expenditure ↓ polysaturated fatty acids	abolished rhythmic expression of clock and clock-output genes in both BAT and WAT loss of circadian variation in plasma TG, glucose	[120]
*Clock*^Δ19^ driven by a*P2* promoter	↑ young mortality rate ↓ rate of glucose tolerance	Not mentioned	[121]
*Bmal1*^−/−^ driven by UCP1	↓ PVAT-induced vasoconstriction	↓ blood pressure during resting phase ↓ angiotensin and angiotensinogen levels in PVAT	[122]

Bmal1, brain and muscle aryl hydrocarbon receptor nuclear translocator-like protein 1; aP2, adipocyte protein 2; HFD, high fat diet; BAT, brown adipose tissue; WAT, white adipose tissue; TG, triglyceride; Clock, circadian locomotor output cycles kaput; UCP1, uncoupling protein 1; PVAT, perivascular adipose tissue.
